# Comparison of Cognitive Function in Children with Stunting and Children with Undernutrition with Normal Stature

**DOI:** 10.1155/2022/9775727

**Published:** 2022-07-12

**Authors:** Setyo Handryastuti, Hardiono D. Pusponegoro, Surastuti Nurdadi, Anita Chandra, Feka A. Pramita, Amanda Soebadi, Ivan R. Widjaja, Achmad Rafli

**Affiliations:** ^1^Department of Child Health, Dr. Cipto Mangunkusumo Tertiary General Hospital-Faculty of Medicine, Universitas Indonesia, Jakarta, Indonesia; ^2^Faculty of Psychology, Universitas Indonesia, Depok, Indonesia

## Abstract

**Background:**

Stunting is the impaired growth and development that children experience from poor nutrition, repeated infection, and inadequate psychosocial stimulation. Children are defined as stunted if their height-for-age is more than two standard deviations below the WHO Child Growth Standards median. According to the Indonesia Basic National Health Survey 2013, Indonesia's stunting prevalence reached 37.2%. Various studies have shown that impaired cognitive development is found in children with stunting and undernutrition. This study aims to determine cognitive development in stunted and undernutrition with normal stature children using the Bayley Scale of Infant Development III (Bayley-III).

**Methods:**

A cross-sectional study on 51 children aged one month to 3 years who fulfilled the inclusion criteria and who visited the outpatient clinic of Dr. Cipto Mangunkusumo National General Hospital from June 2017 to January 2018 was performed. Cognitive development was assessed using the Bayley Scale of Infant Development, Third Edition (Bayley-III).

**Results:**

26 children with stunting and 25 children with undernutrition with normal stature participated in this study. There was a statistically nonsignificant trend toward lower median score percentiles in the stunted group compared to that in the undernourished with normal stature group in the motor (median (range) 1 (0.1–75) vs. 4 (0–79); *p*=0.183), cognitive (12.5 (0.1–75) vs. 16 (0.1–99.9); *p*=0.550), and adaptive behavior (7 (0.1–75) vs. 12 (0.1–58); *p*=0.657) domains.

**Conclusions:**

There is a trend toward lower cognitive, motor, and adaptive behavior abilities in stunted children compared to undernourished children with normal stature which needs further study. In addition, children with undernutrition have below-average abilities across all domains even before stunting has occurred.

## 1. Background

Short stature in children can be caused by several factors, including constitutional factors (e.g., familial short stature, and constitutional delay of growth and puberty), endocrine problems, recurrent or chronic infections, malnutrition, and socioeconomic factors. [1, 2] Stunting, or being too short for one's age, is defined as a height that is more than two standard deviations below the World Health Organization (WHO) Child Growth Standards median [3, 4]. The prevalence of stunting in Indonesia, according to the Indonesia Basic National Health Survey, reached 36.8% in 2007, decreased in 2010 to 35.6%, and increased again to 37.2% in 2013 [5–7]. This undoubtedly will impact the quality of human resources in the future because cognitive disorders may accompany stunting [6, 8].

The association of stunting with long-term child development, including cognitive development, has been reported in various studies. This association warrants serious attention, since the impact of stunting on children's cognitive development may carry over into the next generation. Cognitive impairments cause a further impact on quality of life and require complex multidisciplinary management. Various studies have shown that cognitive impairment may persist even after weight and height have been corrected, and impacted children may require long-term supplementation and cognitive intervention [8, 9]. Before stunting occurs, there are circumstances that precede it, namely inadequate weight gain (weight faltering) and undernutrition. Studies measuring cognitive function in these groups are limited, especially in Indonesia. The question arises whether undernourished children with normal stature are also at risk of cognitive impairment. It is important to address this question in order to identify vulnerable child population groups as targets of public health intervention strategies.

This study aims to measure and compare the cognitive development of stunted children and undernourished children with normal stature aged <3 years using the Bayley Scales of Infant Development, Third Edition (Bayley-III).

## 2. Methods

This was a cross-sectional study involving children aged one month to three years who visited the Dr. Cipto Mangunkusumo National General Hospital's pediatric outpatient clinic between June 2017 and January 2018. Stunted children and undernourished children with normal stature or a history of growth faltering were included in this study. Subjects were categorized as stunted (group 1) or undernourished with normal stature (group 2). The stunted group (group 1) consisted of children with height-for-age *z*-scores of <−2 according to the WHO Child Growth Standards. Children were categorized as undernourished with normal stature (group 2) if their weight-for-age *z*-scores were <−2 and height-for-age *z*-scores were >−2 according to the WHO Child Growth Standards. We excluded children with severe neurological disorders (such as cerebral palsy and congenital neuromuscular disorders), severe neurodevelopmental disorders (such as autism spectrum disorders), and global developmental delay suspected due to toxic disorders, inborn errors or metabolism, or genetic disorders.

All participants underwent anthropometric examination that included weight, height, mid-upper arm circumference, and head circumference, as well as neurologic examination. We classified head circumference as normocephalic (*z*-score ≥ −2 and ≤+2), microcephalic (*z*-score < −2), or macrocephalic (*z*-score > +2) according to the WHO Child Growth Standards growth curve. Subjects who were stunted or undernourished were referred to pediatric endocrinology, nutrition, tropical and infectious diseases, and pulmonology subspecialist clinics for diagnostic evaluation of the possible causes of their respective growth disturbances. Subsequently, cognitive function was evaluated using Bayley-III performed by two trained and experienced child psychologists. Five domains are measured in Bayley-III: cognitive, language, motor, adaptive, and social-emotional skills. Bayley-III scaled scores of 1–6 were classified as below average, 7–13 as average, and 14–19 as above average. Scores were also expressed as percentiles for age and sex [10]. We then compared the scaled scores and percentiles between the two groups.

The minimum number of subjects needed for each group was 20. Statistical analysis was done using the unpaired *t*-test when the data were normally distributed, and using the Mann–Whitney test when the data were not normally distributed. Bayley-III SD scores are calculated between two groups. We used SPSS 21.0 to aid in statistical analysis.

This study was performed in line with the principles of the Declaration of Helsinki. The approval for the study protocol was granted by the Medical Research Ethics Committee of the Faculty of Medicine, University of Indonesia (no. 483/UN2.F1/ETIK/2017). We obtained written informed consent from the parents or guardians of all individual participants included in the study.

## 3. Results

Fifty-one children aged less than three years were included in this study, comprising 26 stunted children (group 1) and 25 undernourished children with normal stature. Subjects in groups 1 and 2 were made up of children who visited the outpatient clinic for a variety of chronic diseases, such as HIV, congenital heart disease, chronic kidney disease, laryngomalacia, chronic liver disease, and chronic diarrhea after intestinal surgery. The mean ages of the subjects in groups 1 and 2 were 11.0 (range 2.0–34.0) months and 13.0 (range 2.0–38.0) months, respectively (Table 1). Most (34/40; 58.6%) were male. Bayley-III SD scores in each domain (cognitive, language, motor, social-emotional, and adaptive) are presented in Table 1.

In all five domains, there was no significant difference in the Bayley-III scores between the two groups. However, scores in both groups were below the 50^th^ percentile in all domains, especially in the motor domain (Table 1).

## 4. Discussion

In this study, we have compared the cognitive function of children with stunting and children with undernutrition with normal stature. Compared to undernourished children with normal stature, the stunted children had slightly lower mean Bayley-III scaled scores in the cognitive, language, motor, and adaptive behavior domains, but the differences were not statistically significant. There was a trend toward notably lower median score percentiles in the stunted group compared to the undernourished with normal stature group in the motor (median (range) 1 (0.1–75) vs. 4 (0–79); *p*=0.183), cognitive (12.5 (0.1–75) vs. 16 (0.1–99.9); *p*=0.550), and adaptive behavior (7 (0.1–75) vs. 12 (0.1–58); *p*=0.657) domains. However, the differences did not reach statistical significance, possibly owing to the wide range of the scores in both groups.

Although not statistically significant, our findings show that further exploration is warranted regarding the developmental and cognitive disadvantages associated with stunting. Moreover, even though children in the undernourished with normal stature group are not stunted, they also have scores below the 50^th^ percentile in all five Bayley-III domains. Our results also suggest the possibility that cognitive impairment may precede stunting.

We included children aged one month to three years as our subjects. This age group was chosen because it represents a period of rapid brain growth susceptible to nutritional deficiencies. At the same time, when cognitive problems are detected during this early age, early intervention can be initiated to mitigate the risk of long-term problems in cognitive development.

The exact mechanisms through which the nutritional status affects cognitive development, as well as the long-term cognitive effects of early malnutrition, remain a subject of exploration to this day. The first 1000 days of life is a crucial period for brain growth and development. Brain volume increases rapidly in children, reaching 60% of adult brain volume at one year and 80% of adult brain volume at two years of age. The brain will reach its final size and cease to grow around 12 years of age [8]. Therefore, adequate nutrition since pregnancy is required for fetal brain development. In addition to brain volume and weight, synapse formation is also accelerated during the first year of life, with children reaching a synapse density comparable to that of adults at two years of age [9].

After birth, especially until the age of two years, synaptogenesis, myelinogenesis, and branching of dendrites and axons will continue to occur. Cognitive function is closely related to the formation of myelin and synapses. Myelin acts as an axon wrapper that speeds up the delivery of information between neuron cells. Synapse is a connection between neuronal cells, forming a gap between neurons where neurotransmitters that regulate brain functions are released. Branching dendrites that function as recipients of information from other neuron cells are also essential [9].

The acceleration of growth and development certainly requires good nutrition both in terms of quality and quantity. Carbohydrates are needed as a source of energy, cellular metabolism, and brain structure formation. Proteins are needed to form hippocampal structures, synaptogenesis (especially essential amino acids), synthesis of growth factors, and cell proliferation and differentiation. Fat is needed for the formation of myelin, synapses, and the visual cortex. Micronutrients (vitamins and minerals) are needed mainly for cell metabolism, synapse, and myelin formation [10].

Malnutrition due to inadequate intake of proteins, carbohydrates, fats, and micronutrients, as well as repeated infections, can cause impaired brain function and structure, tissue damage, growth retardation, impaired cell differentiation, reduced synaptic and neurotransmitter formation, delayed myelination, and an overall reduction in dendritic branching and interferes with the formation of neuronal circuits. Eventually, chronic malnutrition that causes stunting and wasting will result in delays in the development of cognitive processes and permanent cognitive impairment [11].

A study of two groups of children with a history of malnutrition carried out cognitive tests at the age of 5–7 years and 8–10 years. The group with a history of malnutrition showed lower cognitive scores (selective attention using color cancellation test, executive function, visuospatial function, and verbal and visual memory) and language function scores (verbal comprehension and verbal learning) compared to the group with no history of malnutrition. Although the test scores showed slight improvement at the age of eight to ten years, they remained lower in children with malnutrition than in those without. [12] A study involving 5771 infants showed that children who experienced weight faltering from birth to 9 months had significantly lower intelligence quotient (IQ) scores at eight years of age. Weight gain from birth to 8 weeks has a positive correlation with IQ at eight years of age, while the same correlation was not observed with weight gain from 8 weeks to 9 months. This suggests that failure to thrive in early infancy is associated with permanent IQ deficit in later childhood, and the critical period is thought to be between birth and two months of age [13]. A study in Burkina Faso with 532 subjects also showed that children with stunting showed lower neuropsychological scores at age 6–8 years than nonstunted children [14]. These studies have shown that stunting has a lasting impact on cognitive function. In our study, the assessment of cognitive and developmental functions was performed only once. It remains to be seen whether the lack of a significant difference in cognitive function will persist into later life or whether the difference will become more profound as children get older and are required to engage higher, more complex cognitive functions.

Another study showed the lack of fine motor skills in stunted children compared to normal children. Stunted children had lower scores on a test of rapid sequential continuous hand movements than nonstunted children. Even when the stunted children had received nutritional and stimulatory intervention, their test results still showed low scores compared to the group of nonstunted children. Fine motor skills are closely associated with cognitive function and school performance [15]. In the present study, fine motor skills were assessed as part of the motor domain of Bayley-III. We found notably lower score percentiles in the stunted group compared to the undernourished with normal stature group in the motor domain (1 (0.1–75) vs. 4 (0–79); *p*=0.183), as we as the cognitive domain (12.5 (0.1–75) vs. 16 (0.1–99.9); *p*=0.550). These findings, although not statistically significant, may indicate the association between motor abilities and cognitive function.

A longitudinal study of 1674 children studied the effects of early stunting at age 6–18 months and concurrent stunting at age 4.5–6 years on cognitive abilities. It showed that the cognitive abilities of school-age children are associated with early stunting, but have a stronger correlation with concurrent stunting [16]. These results show that interventions to prevent and detect growth faltering in children should continue into school age.

In a case-control study involving 77 subjects with moderate to severe malnutrition in the first year of life in the case group and 59 in the control group, IQ tests were carried out during childhood, adolescence, and young adulthood. The case group showed a lower IQ than the control group at all time points of IQ measurement. This study concluded that moderate to severe malnutrition during infancy is associated with significant cognitive deficits that persist into adulthood, even when physical growth has been corrected. Episodes of malnutrition during the first year of life may result in lifetime cognitive impairment [17–20]. This may be explained by the persistent epigenetic effect of early malnutrition. Through mechanisms such as DNA methylation, malnutrition at an early age can trigger epigenetic changes that persist for decades into adulthood and are correlated with cognitive impairment [18, 21, 22].

The mechanism by which stunting interferes with cognitive function is thought to be related to the low intake of protein. Stunted children have lower levels of essential amino acids than their nonstunted counterparts. Inadequate intake of essential amino acids harms growth because amino acids play a role in protein formation and the synthesis of mammalian target of rapamycin complex 1 (mTORC1), which is essential to the growth of various tissues, including the myelination process [19, 22].

Our study shows undernutrition at an early age may negatively impact all domains of development even though stunting has not yet occurred, as seen from the below-average Bayley-III score percentiles of our group 2 subjects. This can serve to inform public health policymakers that undernutrition in young children less than three years old must be prevented, detected as early as possible, and be subject to intervention before stunting occurs [23].

Our study has several limitations. Subjects were recruited from patients with various chronic diseases seeking treatment at a tertiary referral hospital, so that our results have limited generalizability to children with stunting and undernutrition in the general population. The heterogeneity of underlying conditions was not analyzed in this study, but it may have contributed to the wide variation in Bayley-III score percentiles. We also assessed cognitive function only once; hence, we were unable to evaluate the persistence of the impact of stunting and undernutrition on cognitive function. Larger, population-based, long-term follow-up studies are still needed to obtain more robust data to elucidate the association between stunting with cognitive function.

## 5. Conclusions

Based on our results, we can conclude that there is a trend toward lower cognitive, motor, and adaptive behavior abilities in stunted children compared to undernourished children with normal stature which needs further study. In addition, children with undernutrition have below-average abilities across all domains even before stunting has occurred. Therefore, prevention, early detection, and intervention efforts should be aimed not only toward stunted children, but also toward children experiencing growth faltering.

## Figures and Tables

**Table 1:** Comparison of Bayley-III scores of children with stunting and undernutrition with normal stature.

	Stunting (*n* = 26)	Undernutrition with normal stature (*n* = 25)	*p*
Sex (n)			
Male	16	14	
Female	10	11	

Age (months)	11 (2–34)	16 (9–34)	

Bayley-III score; mean (SD)			
Cognitive	6.5 (1.12)	6.8 (4.19)	0.550^b^
Language	15.3 (6.44)	15.4 (6.12)	0.976^c^
Motor	8.9 (5.26)	10.6 (6.31)	0.298^c^
Social-emotional	8.1 (4.21)	7.5 (4.41)	0.624^c^
Adaptive behavior	58.2 (17.50)	63.7 (16.64)	0.253^c^

Bayley-III percentile; median (range)			
Cognitive	12.5 (0.1–75)	16 (0.1–99.9)	0.550^b^
Language	20.5 (0.1–95)	18 (0.2–94)	0.843^b^
Motor	1 (0.1–75)	4 (0–79)	0.183^b^
Social-emotional	25 (0.4–99)	16 (1–99.9)	0.533^b^
Adaptive behavior	7 (0.1–75)	12 (0.1–58)	0.657^b^

^b^Mann–Whitney test; ^c^unpaired *t*-test.

## Data Availability

The SPSS data used to support the findings of this study are available from the corresponding author upon request.

## References

[1] Fikadu T., Assegid S., Dube L. (2014). Factors associated with stunting among children of age 24 to 59 months in Meskan district, Gurage zone, south Ethiopia: a case-control study. *BMC Public Health*.

[2] Richmond E. J., Rogol A. D., Snyder P. J., Geffner M. E., Hoppin A. G. (2022). Cause of short stature. https://www.uptodate.com/contents/causes-of-short-stature?search=stunting&source=search_result&selectedTitle=3~150&usage_type=default&display_rank=3.

[3] United Nations International (2022). Children’s emergency fund. https://data.unicef.org/topic/nutrition/malnutrition.

[4] World Health Organization (2022). Stunting in a nutshell. https://www.who.int/news/item/19-11-2015-stunting-in-a-nutshell.

[5] Health Research and Development Agency (2008). *Basic Health Research (Riskesdas) 2007*.

[6] Health Research and Development Agency (2010). *Basic Health Research (Riskesdas) 2010*.

[7] Health Research and Development Agency (2013). *Basic Health Research (Riskesdas) 2013*.

[8] Prendergast A. J., Humphrey J. H. (2014). The stunting syndrome in developing countries. *Paediatrics and International Child Health*.

[9] de Onis M., Branca F. (2016). Childhood stunting: a global perspective. *Maternal and Child Nutrition*.

[10] Weiss L. G., Oakland T., Aylward G. P. (2010). *Bayley-III Clinical Use and Interpretation*.

[11] Cherry K. (2020). *The Size of the Human Brain*.

[12] Stiles J., Jernigan T. L. (2010). The basics of brain development. *Neuropsychology Review*.

[13] Georgieff M. K. (2007). Nutrition and the developing brain: nutrient priorities and measurement. *American Journal of Clinical Nutrition*.

[14] Udani P. M. (1992). Protein energy malnutrition (PEM), brain and various facets of child development. *Indian Journal of Pediatrics*.

[15] Kar B. R., Rao S. L., Chandramouli B. A. (2008). Cognitive development in children with chronic protein energy malnutrition. *Behavioral and Brain Functions*.

[16] Emond A. M., Blair P. S., Emmett P. M., Drewett R. F. (2007). Weight faltering in infancy and IQ levels at 8 years in the avon longitudinal study of parents and children. *Pediatrics*.

[17] Sanou A. S., Diallo A. H., Holding P. (2018). Association between stunting and neuro-psychological outcomes among children in Burkina Faso, west Africa. *Child and Adolescent Psychiatry and Mental Health*.

[18] Chang S. M., Walker S. P., Mac Gregor S. G., Powell C. A. (2010). Early childhood stunting and later fine abilities. *Developmental Medicine and Child Neurology*.

[19] Crookston B. T., Dearden K. A., Alder S. C. (2011). Impact of early and concurrent stunting on cognition. *Maternal and Child Nutrition*.

[20] Waber D. P., Bryce C. P., Girard J. M., Zichlin M., Fitzmaurice G. M., Galler J. R. (2014). Impaired IQ and academic skills in adults who experienced moderate to severe infantile malnutrition: a 40-year study. *Nutritional Neuroscience*.

[21] Szutorisz H., Hurd Y. L. (2016). Feeding the developing brain: the persistent epigenetic effects of early life malnutrition. *Biological Psychiatry*.

[22] Semba R. D., Trehan I., Gonzalez-Freire M. (2016). Perspective: the potential role of essential amino acids and the mechanistic target of rapamycin complex 1 (mTORC1) pathway in the pathogenesis of child stunting. *Advances in Nutrition*.

[23] Handryastuti S., Pusponegoro T., Nurdadi S. (2020). Comparison of cognitive and other developmental functions in children with stunting and malnutrition. https://www.researchsquare.com/article/rs-72816/v1.

